# More Than Skin Deep: Autophagy Is Vital for Skin Barrier Function

**DOI:** 10.3389/fimmu.2018.01376

**Published:** 2018-06-25

**Authors:** Payel Sil, Sing-Wai Wong, Jennifer Martinez

**Affiliations:** ^1^Immunity, Inflammation, and Disease Laboratory, National Institute of Environmental Health Sciences, Durham, NC, United States; ^2^Oral and Craniofacial Biomedicine Curriculum, School of Dentistry, University of North Carolina at Chapel Hill, Chapel Hill, NC, United States

**Keywords:** autophagy, skin autoimmunity, selective autophagy, skin diseases, skin cancers

## Abstract

The skin is a highly organized first line of defense that stretches up to 1.8 m^2^ and is home to more than a million commensal bacteria. The microenvironment of skin is driven by factors such as pH, temperature, moisture, sebum level, oxidative stress, diet, resident immune cells, and infectious exposure. The skin has a high turnover of cells as it continually bares itself to environmental stresses. Notwithstanding these limitations, it has devised strategies to adapt as a nutrient-scarce site. To perform its protective function efficiently, it relies on mechanisms to continuously remove dead cells without alarming the immune system, actively purging the dying/senescent cells by immunotolerant efferocytosis. Both canonical (starvation-induced, reactive oxygen species, stress, and environmental insults) and non-canonical (selective) autophagy in the skin have evolved to perform astute due-diligence and housekeeping in a quiescent fashion for survival, cellular functioning, homeostasis, and immune tolerance. The autophagic “homeostatic rheostat” works tirelessly to uphold the delicate balance in immunoregulation and tolerance. If this equilibrium is upset, the immune system can wreak havoc and initiate pathogenesis. Out of all the organs, the skin remains under-studied in the context of autophagy. Here, we touch upon some of the salient features of autophagy active in the skin.

## Introduction

**Figure 1:**
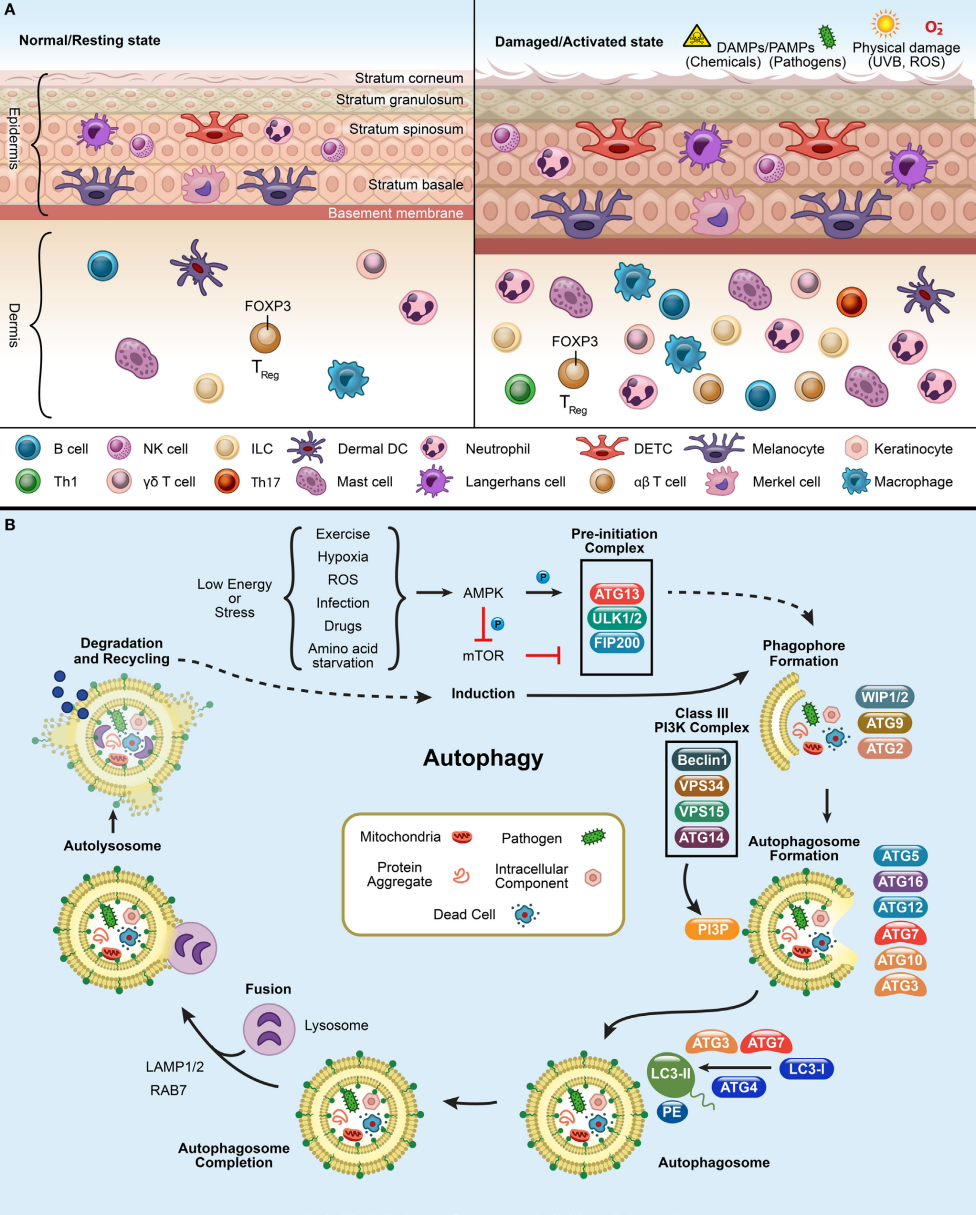
Panel **(A)** depicts the resting and activated state of the skin. In a normal or resting state, epidermis and dermis have circulating immune cells [DETCs, αβ T cells, γd T cells, macrophages, neutrophils, LCs, dermal DCs (dDCs), NK cells, B cells, innate lymphoid cells (ILCs)] and non-immune cells (melanocytes, keratinocytes, and merkel cells). Upon exposure to pathogens, chemicals, UV, or reactive oxygen species (ROS), the immune cells infiltrate at the site of infliction to defend the host and finally to resolve the inflammation after damage. Panel **(B)** shows the process of autophagy. mTOR inhibition triggers the activation of AMPK and initiates an autophagy-inducing signals during a low energy state such as starvation, ROS, exercise, infection, drugs, and hypoxic stress. This initiates the formation of pre-initiation complex (ULK1/2, ATG13, and FIP200) in the presence of unwanted cargo (such as, mitochondria, pathogens, protein aggregates, and intracellular components). This will, in turn activates the Class III phosphatidylinositol-3-kinase (PI3K) complex, composed of ATG14 (UVRAG)-VPS15-VPS34-Beclin1. The Class III PI3K complex completes the autophagosome formation by producing PI3P which recruits downstream ubiquitin-like conjugation systems (ATG5–12) and converts LC3-I to form LC3-PE. Finally, lysosome fuses with the autophagosome to form the autolysosome to degrade the enclosed cargo. The degraded cargo is finally assimilated and recycled.

Skin architecture is designed to shield against physical as well as immunological damage by environmental assaults [such as pathogens, ultraviolet radiation (UVR), allergens, oxidative stress, and various chemical toxins like hexavalent chromium, zinc, titanium oxide, and silver nanoparticles] (1–4). The skin is a nutrient-poor environment, which exposes itself to various environmental stressors regularly and therefore, requires recycling of limited resources *via* the autophagy machinery to maintain homeostasis (5, 6). Nonetheless, skin has a potent arsenal of weapons at its disposal to ward off potential threats from external aggressors. The cells populating the skin have both immune and non-immune components (1, 2). The skin is comprised of the epidermis, dermis, and hypodermis (subcutaneous fat) (Figure 1A) (1, 2, 7, 8). Skin also has several appendages (adena), such as nails, sweat glands, sebaceous glands, and hair follicles, which allow sensation, lubrication, and restriction of heat loss (9). Epidermis is comprised of keratinocytes, Langerhans cells (LCs), dendritic epidermal **γδ** T cells (DETC), melanocytes, and merkel cells (10). Dermis is comprised of fibroblasts, immune cells [dermal DCs (dDCs), innate lymphoid cells (ILCs), NK cells, B cells, macrophages, and T cells], endothelial cells, and neurons, which build up the extracellular matrix (1, 2). The hypodermis is comprised of adipocytes, nerves, blood, and lymphatic vessels.

Apart from the innate and adaptive immune cells present in the skin, the complement systems, antibodies, and antimicrobial peptides (AMPs) aid the immune system in clearing out pathogens and foreign particles. Autophagy participates in various physiological activities to ensure the smooth and quiescent operation of the immunotolerant environment and to maintain skin integrity. These activities include maintaining homeostasis, performing efferocytosis, as well as determining skin color, host defense, longevity, antigen presentation, and survival (11).

## Autophagy as a Cell Survival Mechanism

Autophagy means *self* (auto) *eating* (phagy) and is a highly conserved cellular process across eukaryotes, which allows cells to recycle cytoplasmic materials *via* the lysosome and survive periods of nutrient deprivation (11). The term autophagy is derived from ancient Greek, but the word first garnered attention when Christian de Duve not only coined it but also won the Nobel prize in Physiology or Medicine in 1974 for his work on lysosomes (12–16). More recently Dr. Yoshinori Oshumi, described the autophagy-related genes (ATG) in yeast in 1993 and received the Nobel prize in 2016 (11, 12, 17). His pioneering work led to the discovery of other ATG genes and its human orthologs.

Autophagy pathways include macroautophagy (canonical autophagy/autophagy), microautophagy, and chaperone-mediated autophagy (CMA) (11, 13, 18). Traditionally, autophagy is orchestrated by the group of ATG proteins, which precisely control the autophagic process (11, 19). The process kickstarts the formation of the pre-initiation complex, followed by generation of the phagophore, autophagosome, and autolysosome, leading to cargo degradation (Figure 1B) (11, 19, 20). Mammalian target of rapamycin complex 1 inhibition leads to the induction of autophagy and assembles ULK1/2, ATG13, and FIP200 to form the pre-initiation complex at the phagophore (Figure 1B). Once activated, it targets the Class III phosphatidylinositol-3-kinase (PI3K) complex (Beclin1, VPS34, VPS15, and ATG14) which recruits downstream conjugation ATG proteins. During autophagosome elongation, E3(Ubiquitin)-ligase ATG7 mediates ATG5–ATG12–ATG16L1 complex formation and is recruited to the autophagosome membrane. Ubiquitin-conjugating/E2-like enzyme ATG10 mediates covalent conjugation of the ubiquitin-like ATG12–ATG5 (21). E2-like enzyme ATG3 forms ATG12–ATG3 conjugate, controls mitochondrial homeostasis (21). ATG7 can recruit ATG3 and ATG10 forming ATG7–ATG3 and ATG10–ATG3, respectively (22). Mice lacking ULK1/2, ATG3, ATG5, ATG7, ATG12, or ATG16L1 are embryonic lethal mutations (23). ATG12-conjugation is essential for the formation of preautophagosomes (24). ATG3 aids in conjugation of LC3-I with phosphatidylethanolaminie (PE) required for the formation of autophagosomes (21, 24). This facilitates the LC3 lipidation with PE and forms LC3-PE (or LC3-II). LC3-PE embeds into the mature autophagosome which finally fuses with the lysosome, wherein the cargo is degraded and recycled. The autophagy pathway is not only limited to the processes of degradation and survival during starvation but is also active in regulating other cellular functions (11). This bolsters the need for investigating autophagy’s widespread influence on different biological mechanisms.

To ensure proper scrutiny, the autophagy machinery takes on specialized roles that selectively targets and digests intracellular components and is called selective or non-canonical autophagy (25–27). Depending on the cargo engulfed, it can be classified into CMA (heat-shock cognate 70 stress protein mediated target of the substrate), aggrephagy (clearance of protein aggregates), macrolipidophagy (the degradation of lipids), pexophagy (autophagic degradation of peroxisomes), ER-phagy (endoplasmic reticulum autophagy), mitophagy (damaged mitochondria), xenophagy (intracellular pathogens), and LC3-associated phagocytosis (LAP) (efferocytosis and pathogen phagocytosis) (28–31). Selective autophagy receptors/adaptors p62/Sqstm1 (*Sequestome1*), OPTN (*Optineurin*), TAX1BP1 (T-cell leukemia virus type I binding protein 1), NDP52/CALCOCO2 (calcium binding and coiled-coil domain 2), and NBR1 (neighbor of BRCA1 gene 1) coordinate and mediate degradation of ubiquitinated cargos by delivering them to LC3-containing phagophores (Figure 1B) (11, 32–41). Mitophagy involves degradation of redundant and distressed mitochondria and normally occurs in a Parkin-PINK1-dependent manner (42, 43). After ubiquitination, autophagy adaptors, OPTN and NDP52, can recognize and deliver them to LC3-positive autophagosomes for degradation (44). Similarly, in xenophagy, cytosolic pathogens or pathogen-contained vacuole can be ubiquitinated by ubiquitin ligases (45–47). Subsequently, ubiquitinated pathogens or their substrates are recruited by autophagy receptors for autophagosomal degradation (11). However, when an extracellular pathogen is phagocytosed and it engages pathogen recognition receptor (PRR), as a result it activates a specialized autophagy process called LAP (27). The LAP pathway is also utilized for the clearance of dead cells triggered by wounds, pathogen exposure, or environmental triggers (26, 27, 48).

## Autophagy in Skin Immune Cells

Skin inflammation induced by environmental irritants and pathogens requires autophagy as well as a crosstalk between immune and non-immune skin cells (listed below and in Table 1) to effectively alleviate the damage (4).

**Table 1:** Lists the pertinent autophagy components active in skin cells.

	Cell types	Autophagy mediator	Processes that require the autophagy machinery	Reference
Non-immune cells	Keratinocytes 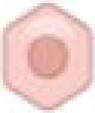	Phosphatidylinositol-3-kinase (PI3K)–AKT–mTOR pathway, ATG5, ATG7	Pigmentation, differentiation, hyperkeratosis	(2, 49–55)

	Melanocytes 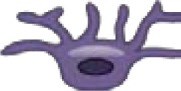	ATG5, ATG7	Aging, oxidative stress damage, UV protection, melanin production	(56–65)

	Merkel cells 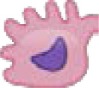	ATG7	Differentiatation, removal of damaged proteins/organelles	(66, 67)

Innate Immune cells	Neutrophils 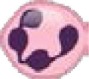	ULK1, Beclin1, ATG16L1	Accelerated apoptosis in leprosy patients, degranulation, reactive oxygen species production	(68, 69)

	Macrophages 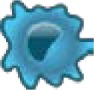	ULK1, Beclin1, ATG14, ATG16L1	Pigmentation, removal of damaged proteins/organelles	(70–73)

	Langerhans cells 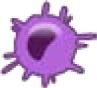	*Maplc3b, Atg3*, ATG7	Removal of melanosomes, control of inflammation, antigen presentation	(74–76)

	Dermal DCs 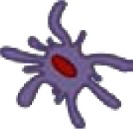	*Maplc3b, Atg3, ATG7*	Removal of melanosomes, control of inflammation, antigen presentation	(74–76)

	Mast cells 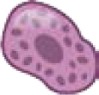	ATG7	Degranulation of mast cells	(77, 78)

	NK cells 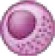	Beclin1	Melanoma	(79)

Adaptive immune cells	B cells 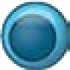	ATG5	Differentation, autoimmune disorders	(80)

	αβ T cells 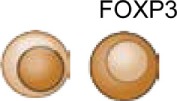	ATG5, ATG7	Inflammation, expansion of T_regs_, and skin homeostasis	(81–85)

	γd T cells 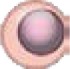	mTOR	Survival and wound repair	(70, 86)

### Non-Immune Components

- *Melanocytes* reside in the epidermis between keratinocytes, and they contain melanin or biochrome, a natural pigment found in hair, skin, and eyes (49). The process of producing melanin pigment continuously is called melanogenesis (87). Hair follicular melanogenesis (FM), unlike epidermal melanogenesis, is cyclic (88). FM starts at the anagen (active growth phase) stage of the hair cycle and is turned off by catagen (transition stage) and telogen (shedding stage) (88). Upon aging, the number of melanocytes in hair follicles is reduced and dendritic cells (including Langerhans cells) move from the upper to lower hair follicles as a response to age-related degradation of melanocytes (88).
  Stress can stimulate melanocyte to activate neurotransmitters, neutropeptides, and hormones which aids in regulating skin homeostasis (89). Neuroendocrine has been shown to govern the production and secretion of l-tyrosine and l-dihydroxyphenylalanine (l-DOPA), or its derivatives, during melanogenisis (89). Melanocytes are often called “neurons of the skin” and possesses melanocytes-stimulating hormone (MSH) receptors (89, 90). Certain substrates of melanogenesis like l-tyrosine and l-DOPA can also regulate cell (melanocyte) functions and cellular metabolism through non-receptor-mediated processes (91).
  Melanocytes develop into melanosomes, which can perform lysosomal degradation as seen in retinal epithelial cells (49,  56, 87). Autophagy is also involved in melanin synthesis in melanosomes (92) *via* FGF7/FGF7R-initiated AMPK/mTOR pathway (93–96). *ATG5* and *ATG7*-deficiency causes premature aging and accumulation of oxidative stress-induced damage (57–60). Upon undergoing UV exposure, melanocytes can undergo photosensitization by generating superoxide radicals in cells (97). In addition, p62 is upregulated upon phototherapy (UVA radiation and light-emitting ode 585 nm) in the melanocyte (61, 62). Melanin confers protection from UV-induced DNA damage, maintains skin homeostasis, modulates the immune environment, and regulates skin color by autophagy (56, 63–65). Melanocytes can also phagocytose, present antigen *via* major histocompatibility complex (MHC) class II and produce cytokines like IL-1, IL-6, TNF-α, IL-3, and G-MCSF (98).
- *Merkel cells* populate broadly across the epidermis. They have high turnover and differentiate terminally (2, 99). Merkel cells have a longer life span than keratinocytes and require autophagy for differentiation (66). *Atg7*-deficient Merkel cells show accumulation of p62 (66, 67).
  *Keratinocytes* are abundant and form the foundation of the epidermis. Out of all skin cells, they have been studied the most. Keratinocytes protect the skin cells by phagocytosing damaged melanosomes (49). Human keratinocytes also induce inflammasomes upon either UVB irradiation or viral infection (100). Autophagy in keratinocytes contributes to skin pigmentation, as it depends on the melanin from phagocytosed melanocytes engulfed by the keratinocytes (49, 92). Keratinocyte differentiation results in lysosomal enzyme activation, LC3 expression, and intracellular components degradation to form corneocytes (2, 50–52). Epidermal permeability barrier, mitophagy, and autophagy defects are observed in comparative gene identification-58 (CGI-58) (a co-activator of adipose triglyceride lipase)-deficient mice (51, 101). CGI-58 deficiency causes Chanarin–Dorfman syndrome (neutral lipid storage dysfunction) and chronic and excessive build up of keratin leading to ichthyosis (51, 101). *Atg7*-deficient keratinocytes are smaller, with outer root sheath thickening, acanthosis, and hyperkeratosis. They also have less keratohyalin, trichohyalin granules, and filaggrin (49, 53, 54, 102). Moreover, *Atg7*-deficient mice have more corneocytes (53). *Atg7*-deficiency leads to cellular aging and accumulation of p53 and p21 upon treatment with N,N′-dimethyl-4,4′-bipyridinium dichloride (paraquat) treatment (54). Keratinocyte growth factor (FGF7/KGF) controls human keratinocyte differentiation and induces autophagy *via* the PI3K–AKT–mTOR pathway (99, 103). Hence, *Atg7*-deficient mice demonstrate the importance of autophagy in epidermal keratinization and hair growth (53, 99, 102). Autophagy inhibition either *via* 3-MA treatment or *Atg5*-deficiency also impairs keratinocytes differentiation (49, 55).

### Immune Components

- *Neutrophils* are a most abundant granulocytes and are considered the foot soldiers of the immune system (68). Autophagy-deficient neutrophils show impaired degranulation, NADPH-oxidase-mediated reactive oxygen species (ROS) production, and inflammatory responses (68). Neutrophils from the skin of leprosy patients release TNF-α, show increased autophagy and exhibit accelerated apoptosis *in vitro* (69).
- *Macrophages* are skin resident phagocytes surveilling the epidermis and dermis (2). They help in maintaining the immunotolerant environment of the skin (2, 92). Specialized macrophages in the skin called melanophages can engulf melanocyte fragments and melanin. These melanocyte fragments and melanin are processed by autophagy (70–72). In leprosy patients, skin-derived macrophages have significant upregulation of autophagy genes, including *Beclin1* and *Atg14* (73).
- *Epidermal DCs, also known as Langerhans cells (LCs)* are dendritic cells of the skin and are found among the keratinocytes in the epidermis (2, 104). The LCs surveil the epidermis and promote tolerance to environmental stressors (104–106). Once activated, LCs migrate to the draining lymph nodes and aid in T cell polarization *via* antigen presentation to define the adaptive immune environment (106). Cytosolic and endocytosed antigens are processing and presentation (or cross presentation) by DCs to either CD4^+^ or CD8^+^ T cells *via* MHC class II and MHC class I molecules, respectively, and this process requires the autophagy machinery (74). Macroautophagy (canonical autophagy) has been implicated in the intracellular antigen loading on MHC class II molecule, allows the autophagosomal membrane to fuse with the MHC class II-loading compartment and thus, allows efficient MHC class II presentation (75). Mintern et al. showed that CD8^+^ splenic DCs from *ATG7-deficient* mice have impaired cross presentation of antigen *via* MHC class I pathway but can efficiently load antigen on MHC class II molecule (74). Thus, suggesting a role of autophagy in antigen presentation and cross presentation by DCs.
  Both LCs and *dDCs* can induce interferon (IFN) and pro-inflammatory responses (107). Type I IFN can induce autophagy during viral infection, autoimmune disorders, and certain cancers (108, 109). ATG, *Map1lc3b* and *Atg*3 have been shown to be important in the production of hapten 2,4-dinitrofluorobenzene-induced (DNFB) ROS in skin DCs (76).
- *Mast cells* are granulocytes crucial for allergic inflammatory reactions (77). In the skin, they are triggered by UV or ROS-inducing irritants and undergo degranulation (77, 78). In addition, the degranulation process requires ATG7 (77).
- *NK cells* are cytotoxic innate immune lymphocytes present in the dermis. A recent study showed that targeting autophagy by inhibiting Beclin1 can increase NK cells resulting in melanoma tumor (79). Beclin1 inhibition reduces degradation of NK-derived Granzyme B as a result of which melanoma cells can thrive even in the presence of NK cells driven by CCL5 (79).
- *ILCs* are a new addition to the family of innate immune cells, and they are involved in shaping the immune system (110, 111). There are three types of ILCs defined by their cytokine profile: ILC1 produces IFN-γ, ILC2 produce IL-5 and IL-13, and ILC3 produces IL-17A and IL-22 (110). In human and murine skins, ILC2 are the most prelevant. The role of ILCs in skin autophagy is not well reported.
- Human skin contains approximately 20 billion skin *T cells or T lymphocytes* (112). *T cells* define the adaptive immune responses and are differentiated from other lymphocytes by the presence of surface T cell receptor (TCR) (112). TCR engagement activates autophagy, induces ROS, and causes activation of nuclear factor of activated T-cells (NFATC1) and lysosome-associated membrane protein 2 (LAMP-2) (113). Autophagy can alter the T cell repertoire through selection and aid in cross presentation by DCs (113–116).
  ✓ *Alpha beta (*αβ*) T cells* (CD8^+^ T cells and CD4^+^ T cells) survive and persist in the skin long after the immune reaction is over (113, 117–123). This ensures rapid protection from future exposures to pathogens or antigens (112, 124). Skin resident-memory T_reg_ cells mitigate inflammation and regulate immune response *via* autophagy (81, 82). T_regs_ (20–60% of CD4^+^ T cells) have also been shown to alleviate autoimmune disorder by suppressing DC autophagy (81, 83). *Atg7*-deficient T_regs_ cannot establish skin homeostasis (84, 85).
  ✓ *Gamma delta* (*γd) T cells* comprise 1–5% of the circulating T cells found in both mice and humans (125). However, in skin about 50% of γd T cells are present among the T cells (126). They populate the skin epidermis and dermis and are named dendritic epidermal γd T cells (DETCs) and dermal γd T cells, respectively (125, 127). Like αβ T cells, they possess effector functions as well as mediate tissue repair (86, 125). γd T cells are the first responders to skin damage and are responsible for maintaining homeostasis, wound repair, and production of cytokines like insulin-like growth factor 1, TNF-α, and KGF-1 (86). DETCs are in close contact with keratinocytes and aid in wound healing by releasing KGF-7 and KGF-10 (128, 129). Skin γd T cells are adaptable under immunosuppression by rapamycin (inhibits mTOR pathway) or roseotoxin B (suppresses activated T cells but does not effect naïve T cells), and they undergo autophagy to survive in the absence of cytokines (70, 86).
- *B cells* are a component of the adaptive immune system populating the dermal layers and produces antibodies. In B cells, ATG5 seems to play an important role in differentiation and survival (80). Autoantibodies induce autophagy and can sometimes lead to autoimmune disorders (80).

## Skin Exposures and Autophagy

When the skin encounters pathogens, injury, or UVR, it deploys various defense mechanisms. Autophagy responds to these unwanted encounters to ensure inflammation resolution. Defects in autophagy can cause a hyperinflammatory skin reaction due to inflammasome activation (as seen in human keratinocytes), unpredicted ROS activation *via* UVR, and aberrant pro-inflammatory cytokine release (Figure 1A) (4, 113, 130–133).

### Pathogens

Evolutionarily, epidermal pathogenic bacteria have devised multiple evasion mechanisms to avoid autophagic clearance (134, 135). Numerous instances of the autophagy machinery engaging skin pathogens during cutaneous infection have been described (Table 2). Autophagy is critical for the clearance of group A *streptococci* (GAS) which evades endosomal capture (13, 136). A recent study showed that the elimination of GAS is severely reduced in *Atg5-deficient* cells elucidating its vital role in pathogen clearance (13, 136). *Streptococcus pyogenes* is responsible for causing impetigo, a common skin infection in children (137, 138). Similarly, autophagy is essential in preventing infectious skin diseases such as *Staphylococcus epidermidis* infection, leprosy, and sepsis (Table 1).

**Table 2:** Showing the different autophagy markers involved in skin-related diseases.

Disease	Autophagy markers and associated skin cells	Reference
**Autoimmune disorders and cancer**		

Psoriasis	↓AP1S3 (keratinocytes), ↓ATG16L1 (keratinocytes)	(13, 139–141)

Systemic lupus erythematosus pathogenesis	↓ATG5 (human keratinocytes), ↓UVRAG (human keratinocytes)	(13)

Vitiligo	↓UVRAG (melanocytes), ↓Nrf2 (melanocytes)	(142–144)
	↑p62 accumulation (melanocytes), ↑unfolded protein response, ↑autophagy induction (melanocytes), Atg7↓(melanocytes)	

Diabetic skin disease/ulcer	↑Autophagy *via* AGEs (murine M1 macrophages)	(145–148)

Allergic contact dermatitis	↑Autophagy (murine skin)	(70)

Chanarin–Dorfman syndrome	↓Mitophagy (PINK1), ↓autophagy (murine keratinocytes and human skin)	(51, 101)

Merkell cell carcinoma (MCC)	↑mTOR, ↑p62 accumulation (human MCC cell lines)	(149–151)

Melanoma/malignant melanoma	↓*Beclin1*, ↓*Atg5*, and ↓*Atg7* [human melanoma cell lines (WM35, WM793, 451LU, A2058, and A375), SK-Mel-cell lines, and melanocytes]	(13, 152–161)
	miR-638 blockade—↑autophagy and ↑metastasis	
	Hydroxychloroquine-mediated autophagy inhibition therapy in clinical trials	
	↑miR-23a-ATG12 axis results in ↓ melanoma metastasis	

Infectious diseases		

*Staphylococcus epidermidis* (*S. aureus*)	Autophagosomal degradation diminishes bacterial accessory gene regulator activity (Hela cells and murine bone marrow-derived DCs)	(162)

Impetigo (*group A streptococci or Streptococcus pyogenes*)	↓ATG5, streptococcal cysteine protease (SpeB)-mediated ↓ubiquitin-LC3 adaptor proteins (HEp-2 epithelial cells)	(163)

Sepsis (MRSA)	↑Keratinocyte autophagy, ↓inflammasome	(164–167)
	↑ATG16L1 [human keratinocytes, murine ear skin, HAP1 fibroblast cells, ATG16L1 hypomorphic mice (*ATG16l1*^HM^)]	

Leprosy (*Mycobacterium leprae)*	↑BECN1, ↑ATG14, ↑LC3	(73)
	SQSTM1/p62, ↑NBR1, ↑autophagy by interferon-γ [skin macrophages (dead *M. leprae* induces autophagy and live *M. leprae* reduces it)]	

Cutaneous candidiasis/*Candida* intertrigo (invasive form) (*C. albicans*)	↓ATG5 (murine macrophages), ↓LC3-associated phagocytosis (murine macrophages)	(25, 168)

Warts [human papillomavirus (HPV)]	↑ATG5 (HPV16 infected primary human keratinocytes), ↑ATG7 (HPV16 infected primary human keratinocytes)	(99)
	Autophagosomal degradation of viral capsid proteins (HPV16 infected primary human keratinocytes)	

Oral and genital herpes simplex [herpes simplex virus (HSV)-1 and HSV-2]	↑Autophagy, ↑viral antigen processing and presenting (murine macrophages and dendritic cells), ↓ATG5 (HSV-2 infection in murine fibroblast, human foreskin fibroblasts), ↓Beclin1 and ↓autophagy (by HSV-1-encoded neurovirulence protein, ICP34.5)	(13, 169–171)

Chicken pox (VZV)	↑Autophagy induction (human keratinocytes) to protect	(172–174)

Zika virus infection (ZIKV)	↓Akt-mTOR signaling, ↑autophagy, ↑ATG16L controls ZIKV infection (fibroblasts, keratinocytes and skin DCs, pregnant *ATG16l1*^HM^ mice)	(175, 176)

The skin is susceptible to fungal infections, such as cutaneous candidiasis and *Candida* intertrigo caused by *Candida albicans* (168). Studies have shown that the intracellular clearance of *C. albicans* depends on both autophagy (ATG5) and LAP (168, 177). Autophagy in skin also plays a unique role in anti-viral function (Table 2). Herpes simplex virus (HSV) is a double-stranded DNA virus. It is categorized into HSV-1 and HSV-2, which causes oral herpes and genital herpes (13). Autophagy aids in processing and presentation of HSV-1 antigens on MHC class I molecule for effective viral elimination (178–180). HSV-2 is more susceptible to the host ATG5 (13, 179, 181). Furthermore, skin-related viruses, including human papillomavirus, varicella zoster virus, and Zika virus can induce autophagy to degrade viral capsid proteins in the skin cells and keratinocytes (172, 182).

### Wound Healing

During wound healing after an injury or pathogen invasion, skin immune responses halt the ongoing inflammation to initiate the restoration process (2). In rats, autophagy heals burnt hair follicle epithelium (183). A recent study shows that the use of mesenchymal stem cells (MSCs) in skin repair requires autophagy (184). Rapamycin-induced autophagy in MSCs causes secretion of vascular endothelial growth factor (VEGF) and improves VEGF-mediated blood circulation which in turn promotes skin wound healing and tissue regeneration (184).

### Ultraviolet Radiation

Sun-generated UVR is a mixture of UVA and UVB (185). Upon exposure to UVR, basal autophagy increases in keratinocytes (28) and causes epidermal thickening or hyperkeratosis (28). This in turn, leads to epidermal hyperplasia which prevents the UVR (UVA or UVB, depending on the wavelength) to penetrate the skin (28, 186). UVR-induced cell death in the skin can promote autoimmunity due to defective clearance of apoptotic keratinocytes (5, 108). Patients with systemic lupus erythematosus, an autophagy-related autoimmune disorder, are unable to clear dead cells and suffer from severe cutaneous lesions upon exposure to UVR as a result (187–191). UVR also inhibits antigen presentation by LCs and T_reg_ migration, as seen in cutaneous T cell-mediated dermatitis (70, 192–196).

UVA (long wavelength) irradiation penetrates the dermis, and its exposure induces autophagy to remove p62-associated protein aggregates in keratinocytes and melanocytes (5, 61, 197, 198). Chronic UVA exposure causes apoptosis of epidermal and dermal cells, photoaging, and skin pathogenesis, depending upon the presence of melanin (28, 185). Luteolin (3, 4, 5, 7-tetrahydroxyflavone), a flavonoid showing anti-cancer properties, has recently been shown to decrease UVA-induced autophagy in human skin fibroblasts by scavenging ROS (95, 191).

UVB irradiation entry is limited to the epidermis (198–200). UVB-induced autophagy involves glycogen synthase kinase signaling, which helps protect the epidermal cells from UVB-induced apoptosis (201). UVB-induced ROS downregulates mTOR in skin epidermal cells and induces autophagy (28, 187, 202). Thus, ROS inhibition prevents T cell-mediated dermatitis in mice (203). UVB-treated murine splenocytes show systemic immunosuprression by inhibiting both IFN-γ and IL-10 cytokine production 24 h post-irradiation (204).

UVB radiation also stimulates AMPs (psoriasin, RNase 7, human β-defensin [HBD 1–4]) in human keratinocytes (7, 8, 205). To protect against UVB-induced DNA damage, oxidative stress, cancer, and skin cells produce fat soluble vitamin D (206). Epidermal AMPs like cathelicidin (hBD18) expressed in keratinocytes is induced by 1,25 (OH)_2_ vitamin D3 from 7-dehydrocholesterol and protects the skin against pathogens (8, 206, 207). Vitamin D promotes autophagy (ATG16L1 in autoimmune disorders like inflammatory bowel disorder) and suppresses pro-inflammatory pathways (such as p38 MAPK-mediated signaling pathway, prostaglandin pathway, nuclear factor kappa B signaling pathway) (206, 208, 209).

UV stimulates the central stress response center *via* hypothalamic–pituitary–adrenal axis, however, the mechanism is not well understood (204). UVR-induced β-endorphin and corticotropin-releasing hormone release from the skin causes soluble neuro-endocrine-immune factors to seep into the systemic circulation (204, 210). The skin immune cells stimulated by UV act as “second messengers” allowing crosstalk between neuroendocrine system and immune system (210). Certain neuropeptides, such as calcitonin gene-related peptide (CGRP), mediate anti-inflammatory environment by UVR (211). Steriods like proopiomelanocortin produced from α-melanocyte-stimulating hormone (α-MSH) of UVR-triggered epidermal, dermal cells and macrophages induce immunosuppression (210, 211).

## Skin Cancer and Autoimmune Disorders

Autoimmune disorders are the result of the adaptive immune system generating autoreactive lymphocytes (T and B cells) that target self-antigens. Skin disorders often arise as a secondary complication, as reported in diabetes, cancer, and dermatitis. The role of autophagy reported in skin-related disorders (Table 1) is limited, yet nonetheless deserves recognition.

*Atg5*-deficiency, *Atg7*-deficiency, and *Beclin-1* partial deletion can spur spontaneous tumor growth commonly seen in most cancers (152, 212–214). Contrary to that, targeting Beclin-1 inhibits autophagy, overexpresses CCL5 and aids in recruit NK cells to the melanoma tumor (79). Melanoma is fatal in its aggressive form and is highly metastatic (153). *Atg5*-deficient melanoma cells *in vitro* have diminished survival (152, 154). Interestingly, in advanced stages, melanoma cells promote a tumor-suppressive environment by hijacking the autophagy machinery to ease stress induced by drugs (215). This suggests that cancer cells can manipulate the autophagy machinery to resist treatment (154, 216).

Diabetes mellitus (both type 1 and type 2) patients have defective insulin signaling in the keratinocytes and often suffer from skin lesions and foot ulcers (145–147, 217). In diabetic patients, IRF8 (an autophagy regulator) activation induces autophagy, poises the macrophages to permit inflammation and thus, impairs cutaneous wound healing (145, 217). Chronic hyperglycemia and hyperlipidemia disrupt ER homeostasis and resulted in increased unfolded protein burden (147, 217). Both autophagy and mitophagy are defective in diabetic patients and inhibit keratinocyte proliferation and migration that are requisite for wound healing (145–147).

Psoriasis is a chronic, polygenic autoimmune disease characterized by epidermal hyperplasia, defective keratinization, and infiltration of immune cells within the skin, causing dermatitis and thickened plaques formation. Genetic screening of psoriasis patients of Estonian origin reveals several single-nucleotide polymorphisms (SNPs) associated with *ATG16L1*, though the functional role of ATG16L1 variants in skin biology is unclear (139, 218). In pustular psoriasis cases, mutations in *AP1S3*, a gene encoding an autophagosome trafficking protein, result in the disruption of autophagy in keratinocytes and drive them to produce pro-inflammatory cytokines, including IL-1β, IL-8, and IL-36A (140). Furthermore, a recent study demonstrated that autophagy inhibition *in vivo* shows aberrations in keratinocyte differentiation, resulting in dysregulation of autophagy in psoriatic epidermis (219).

Vitiligo is a pigmentation disorder characterized by sharp demarcated white macules on skin due to the CD8^+^ T cell-mediated destruction of melanocytes (220, 221). This results in localized (segmental vitiligo) and/or generalized (non-segmental vitiligo) partial loss of melanin. In addition, melanocytes show increased autophagy due to misfolding of *tyrosinase (tyr)* and *X-box binding protein 1 (Xbp1)* in the ER (220, 221). Individuals suffering from vitiligo may have defective epidermal permeability barrier functions which can be alleviated by the use of topical histamine treatment (222, 223). Vitiligo patients from Chinese Han population show abnormal antioxidant *Nrf2* [nuclear factor (erythroid-derived 2)-like 2] gene expressions in autophagy and associated pathways (142, 224, 225). Compared to normal melanocytes, vitiligo melanocytes *in vitro* show greater similarity with *Atg7-deficient* melanocytes (57, 60). Both exhibit impaired redox-sensitive Nrf2 activation and decreased activation of the antioxidant enzyme system in response to oxidative challenges induced by environmental stress and ROS (93, 226, 227). Multiple evidences indicate that autophagy controls melanosome degradation (92, 228). Furthermore, a Korean cohort study demonstrated an association between two *UVRAG* gene SNPs and non-segmental vitiligo (vitiligo vulgaris) (229). Skin autoimmune disorders are overly complicated, involve many aspects of the immune system and arise due to defects in autophagy machinery. Accumulating evidence displaying the importance of autophagy in skin disorders demonstrates the need for further research.

## Concluding Remarks

Being at the forefront of the environmental defense system, a plethora of specialized cells reinforces the skin. It is a unique surface that is highly specialized in preserving immunotolerance. Therefore, autophagic clearance of senescent and damaged cells is necessary for the maintenance of specialized cellular machinery to effectively keep inflammatory triggers at bay. Several research groups have implicated the role of ATG5, ATG7, and ATG16L1 in keratinocytes, melanocytes, and immune cells in autoimmunity and cancer. Skin in the context of autophagy remains an uncharted territory and warrants further investigation. Autophagy dictates the immune response and resolution in skin cells to neutralize pathogens, clear senescent cells, or heal wounds. However, the autophagy machinery can either protect or cause autoimmune disorders. The physiological circumstances that govern this “balancing act” are not well understood. The cytokine microenvironment from dying immune cells, pathogens, or senescent cells can potentially direct the autophagic response in skin. In addition, there are evidences suggesting potential communication between autophagic machinery and skin cells (both immune and non-immune cells) in an inflammatory state that resonates in the literature; however, the mechanism is not yet reported (230). Adequate tools to examine the molecular response are lacking.

In addition, skin microbiota has been shown to shape the immune response by producing AMPs, complement, IL-1, and IL-17 as well as modulating the local T cell response (3, 231). The interaction with skin cells and microbiota may reveal underlying mechanisms to aid in conscientious operation of the autophagic machinery as seen in Crohn’s disease and colorectal cancer (232–234). Overall, further research investigating the role of autophagy in barrier function will pave the way forward for therapeutic advancements in the field of skin inflammaging and dermatology.

## Author Contributions

PS and JM outlined the structure and theme of the paper. PS and S-WW performed the systemic literature search. S-WW helped with the disease section and table 2 of the manuscript. PS developed and wrote the final version manuscript. PS also performed the revisions suggested by the reviewers. JM provided valuable guidance while formulating the manuscript and supervised the work.

## Conflict of Interest Statement

The authors declare that the research was conducted in the absence of any commercial or financial relationships that could be construed as a potential conflict of interest.
